# Serum ApoB levels in depressive patients: associated with cognitive deficits

**DOI:** 10.1038/srep39992

**Published:** 2017-01-05

**Authors:** Li Hui, Mei Han, Xiang Dong Du, Bao Hua Zhang, Shu Chang He, Tian Nan Shao, Guang Zhong Yin

**Affiliations:** 1 Institute of Mental Health, Suzhou Psychiatric Hospital, The Affiliated Guangji Hospital of Soochow University, Suzhou 215008, Jiangsu, PR China; 2 School of Medicine, IHMRI, University of Wollongong, Wollongong, NSW 2522, Australia; 3 Beijing HuilongGuan Hospital, Peking University, Beijing 100096, PR China; 4 Department of Psychology, Peking University, Beijing 100000, PR China

## Abstract

Cognitive deficits have been regarded as one of the most significant clinical symptoms of depressive disorder. Accumulating evidence has shown that apolipoprotein B (ApoB) levels, which are responsible for inducing neurodegeneration, may be involved in cognitive deficits. This study examines cognitive deficits, and the correlation of serum ApoB levels with cognitive deficits of depressive disorder. 90 depressive patients and 90 healthy controls with matched age and gender were recruited. Cognition was assessed using the Repeatable Battery for the Assessment of Neuropsychological Status (RBANS). Serum ApoB levels in depressive patients were measured by immunoturbidimetric method. Our results showed that depressive patients had lower scores of cognition including RBANS total score and subscales of language and delayed memory (all, p < 0.001) than healthy controls after controlling for the variables. The differences in cognitive functions also passed Bonferroni corrections. Serum ApoB levels were negatively correlated with delayed memory score in depressive patients (r = −0.30, p = 0.01). Furthermore, stepwise multivariate regression analysis indicated that serum ApoB levels independently contributed to delayed memory in depressive patients (t = −2.68, p = 0.01). Our findings support that serum ApoB levels may be involved in delayed memory decline in depressive patients. Depressive patients also experience greater cognitive deficits, especially in delayed memory and language than healthy controls.

Depressive disorder is one of the most common psychiatric disorders affecting millions of people worldwide^1^,^2^, and is closely associated with more frequent relapse, higher burden of illness, higher risk for suicide, and poorer quality of life^3^,^4^. Although depressive disorder mainly involved in mood disturbance, cognitive deficits have now been a well-established feature of this disorder^5^,^6^. The meta-analysis studies have indicated that cognitive deficits in depressive patients mainly appeared in the following domains, such as attention, memory, processing speed, executive functioning, and selective cognitive control^7^,^8^,^9^. Cognitive deficits in depressive patients were found to further lead to increased disability and mortality, and shortened life time^10^,^11^,^12^,^13^. Cognitive deficits should thus be considered a critical clinical and therapeutic target of depressive disorder. Furthermore, cognitive deficits in differing stages of depressive disorder have been reported. Recent studies have shown that cognitive functions in first-episode drug-free depressive patients were impaired in a Chinese population^14^,^15^. Current depressive patients have been found to have lower scores of all domains of cognitive functions than healthy controls, and there were significant differences in immediate memory and attention between previous depressive patients and healthy controls^16^. Other studies have revealed that cognitive functions significantly declined in recurrent depressive patients with each successive episode of depression^17^,^18^. Cognitive deficits frequently persisted despite the remission of depressive episodes^19^. These studies have provided evidence that cognitive deficits in depressive patients may be pervasive and long-lasting. However, the etiology of cognitive deficits in depressive patients remains uncertain, which should be further investigated.

Serum apolipoprotein B (ApoB) is synthesized in the liver and small intestine rather than the brain, and is used for cholesterol transport in periphery^20^. Several human studies have indicated serum ApoB levels may be involved in cognitive dysfunction. A recent study has showed higher serum ApoB levels in older individuals with amnestic mild cognitive impairments compared to healthy controls^21^. The variables of serum ApoB levels have been reported to be associated with cognitive changes prior to age 65^22^. Intriguingly, a post-mortem study has found that higher ApoB levels were significantly associated with higher brain Abeta43 levels that were involved in the dysfunctions of verbal learning, delayed verbal recall and response inhibition in neurodegenerative disorders^23^,^24^. Several animal studies also have shown a significant association between ApoB and cognitive deficits. For example, ApoB overexpression in hAPPs1 mice alone induced memory decline compared to those in wild-type mice^25^. The addition of the ApoB transport domain to the secreted neprilysin was reported to be able to improve memory deficits in amyloid protein precursor transgenic model of Alzheimer’s disease^26^. These findings suggest that serum ApoB levels may influence cognitive functions in human and animal. However, no studies have examined the correlation between serum ApoB levels with cognitive deficits of depressive disorder. The purpose of the present study is to examine whether depressive patients have worse cognitive functions than healthy controls, and to further investigate whether serum ApoB levels contribute to cognitive deficits of depressive disorder.

## Results

### Clinical and Demographic Characteristics

Table 1 showed no significant difference in gender, age, and education between depressive patients and healthy controls (all p > 0.05). However, there were significant differences in body mass index (BMI) (F = 23.72, p < 0.001), smoking status (χ^2^ = 11.75, df = 1, p < 0.001), and suicide status (χ^2^ = 81.29, df = 1, p < 0.001) between two groups. The mean and standard deviation (Mean ± SD) of age of illness onset, age of first hospitalization, self-rating depression scale (SDS) and self-rating anxiety scale (SAS) standards score, and serum ApoB levels in depressive patients were 31.30 ± 10.23 years, 33.67 ± 10.54 years, 61.77 ± 12.73, 52.00 ± 12.14, and 0.86 ± 0.21 g/L. They had duration of illness for an average 50.70 ± 90.31 months, with hospitalization number for a mean of 0.96 ± 0.92 time. The types of antidepressants included serotonergic and noradrenergic reputake inhibitor (SNRI, 22.22%), selective serotonergic reuptake inhibitor (SSRI, 58.89%), and never taking antidepressants (18.89%).

### Main Findings

The Mean ± SD of the Repeatable Battery for the Assessment of Neuropsychological Status (RBANS) total and index scores of cognitive functions in 90 depressive patients and 90 healthy controls were shown in Table 2. There were significant differences in cognitive functions including RBANS total score (75.17 ± 15.15 vs. 84.73 ± 13.15, F = 20.46, df = 1, p < 0.001) and subscales of language (76.70 ± 15.25 vs. 96.36 ± 13.57, F = 83.42, df = 1, p < 0.001), and delayed memory (77.43 ± 19.02 vs. 95.97 ± 54.73, F = 9.21, df = 1, p = 0.003) between depressive patients and healthy controls. All these differences remained significant with low p values for RBANS total score (F = 19.56, df = 1, p < 0.001) and subscales of language (F = 58.21, df = 1, p < 0.001), and delayed memory (F = 7.72, df = 1, p = 0.006) between two groups after controlling for gender, age, education, BMI, smoking, and suicide status using MANOVA (Table 2). Furthermore, significant differences in RBANS total score and subscales of language, and delayed memory between two groups also passed Bonferroni corrections (all p < 0.05) (Table 2).

**Figure 1:**
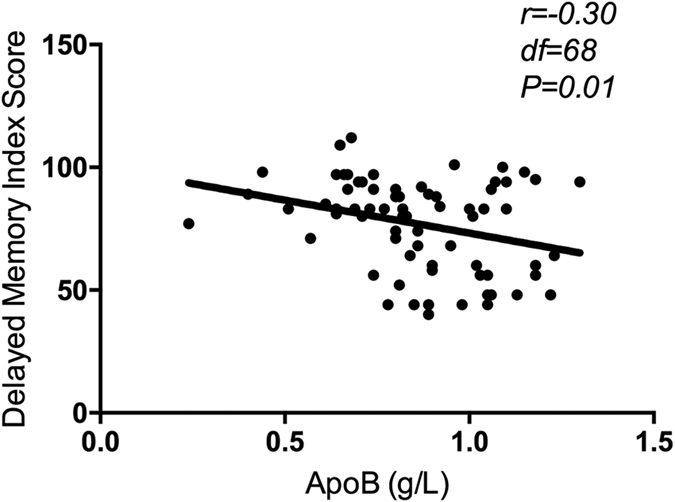
Significantly negative correlation between Apo B and delayed memory index score in depressive patients (r = −0.3, df = 68, p = 0.01).

For depressive patients, correlation analysis showed a significant negative association between serum ApoB levels and delayed memory index score (r = −0.30, df = 68, p = 0.01) (Fig. 1). Further stepwise multivariate regression analysis showed that serum ApoB levels (ß = −27.42, t = −2.68, p = 0.01), education (ß = 1.83, t = 2.55, p = 0.01), and age of illness onset (ß = −1.33, t = −2.21, p = 0.03) independently contributed to delayed memory score, which together accounted for 23.3% of the variance in delayed memory score of depressive disorder. However, serum ApoB levels were not found to be associated with RBANS total score and any other cognitive index scores in depressive patients (all p > 0.05).

## Discussion

To the best of our knowledge, this is the first study to investigate the relationship between serum ApoB levels and cognitive functions in depressive patients. We find the effect of higher serum ApoB levels on delayed memory decline of depressive disorder. Depressive patients also have poorer cognitive functions than healthy controls, especially in delayed memory and language.

This study found cognitive functions were significantly impaired in depressive patients, which was consistent with other clinical studies that depressive patients experienced greater cognitive deficts^5^,^27^,^28^. Our finding was supported by several meta-analysis studies^7^,^8^,^9^. Another study adopting RBNAS for cognitive performance has shown that RBANS total score and five subscales of cognitive functions were significantly lower in depressive patients than healthy controls^16^. However, our results showed that there were significant differences in RBANS total score and subscales of delayed memory and language between depressive patients and healthy controls. The inconsistent findings between two studies could be due to demographic and clinical information differences, and ethnic background.

Previous studies have indicated that hippocampal formation could play a critical role in the regulation of cognitive functions^29^,^30^. A study has also found that reduced hippocampal volumes were associated with memory loss of depressive disorder^31^. The variables of hippocampus have been reported to contribute to language beyond its traditional role in memory in several recent studies^32^,^33^,^34^,^35^. These findings suggest that abnormal hippocampal formation may be related to cognitive deficits of depressive disorder. Therefore, a further study on brain imaging-cognition in depressive patients should be performed, focusing on language and memory.

Interestingly, this study was the first to find that serum ApoB levels were negatively correlated with delayed memory decline of depressive disorder. The underlying mechanisms responsible for this could be that serum high ApoB levels are related to the dysfunctions of lipid peroxidation, malondialdehyde (MDA) and immune system that could been caused by oxidative stress and free radicals of depressive disorders that is associated with cognitive decline^36^,^37^,^38^,^39^. Elevated levels of MDA have been reported to adversely affect the efficiency of delayed memory in patients with depressive disorder^40^. The postmortem and blood studies have shown that oxidative stress and cytokines played an important role in patients with depressive disorder^41^,^42^,^43^, which influenced cognitive functions^44^. Several previous postmortem studies have indicated that oxidative stress was involved in the etiology of neurodegenerative disorders, which was significantly associated with cognitive decline^45^,^46^,^47^,^48^,^49^,^50^. Furthermore, serum ApoB change was significantly correlated with the decline of cognitive performance in older individuals with mild cognitive impairments^36^,^51^. Serum ApoB levels have been reported to be positively associated with brain Abeta43 levels which influenced delayed memory of neurodegenerative disorders^23^,^24^. ApoB overexpression has been found to induce memory decline in hAPPs1 mice^25^. The fusion protein of ApoB transport domain and neprilysin was involved in improving memory deficits in animal model of Alzheimer’s disease^26^. These results support that serum ApoB levels, which are influenced by oxidative stress and free radicals, may contribute to delayed memory decline of depressive disorder.

Several limitations in this study should be noted. First, the association between serum ApoB levels and delayed memory in depressive patients was investigated by correlation and stepwise multivariate regression analyses. Therefore, the explanation of causal relationships was rather caution. Second, our samples were collected from Suzhou area, thus our findings may not be completely applicable to depressive patients in other cities of China. Finally, some other clinical information including residual symptom, recurrent episodes and remission status were not collected, which should be considered in statistical analysis as they can influence cognitive functions in depressive patients. The above factors could lead to deviation of our results. Therefore, a strictly designed study may be performed in the future to confirm our results.

In summary, we found that serum ApoB levels might play a crucial role in delayed memory decline in depressive patients. Depressive patients experienced greater cognitive deficits than healthy controls, especially in delayed memory and language. However, this study should be viewed as a preliminary investigation, and further studies in larger, independent samples with case-control matching in different ethnicities are warranted to exclude the possibility of false-positive findings.

## Methods

### Study Population

This study was conducted between September 2014 and February 2016. Depressive patients (n = 90; male/female = 30/60) were recruited from the inpatient unit and outpatient clinic of the Affiliated Guangji Hospital of Soochow University. The catchment area of this hospital covered a population of approximately 9,060,000. Patients who met the following criteria were recruited to participate in the study: (a) age between 17 and 65 years; (b) diagnosis of unipolar depression according to the Diagnostic and Statistical Manual of Mental Disorders, Forth (DSM–IV); (c) received education for at least 4 years; and (d) provided written informed consent and were able to take part in the cognitive assessment. Diagnoses were performed for each patient by two independent experienced clinical psychologists and confirmed using the Structured Clinical Interview for DSM-IV.

Healthy controls (n = 90; male/female = 30/60) were recruited from the employees of the Affiliated Guangji Hospital of Soochow University. Current mental status and personal or family history of mental disorders were assessed using unstructured interviews. None of the healthy controls presented a personal or family history of psychiatric disorders.

Depressive patients and healthy controls with matched age and gender were Han Chinese from the Suzhou area. All subjects were in good physical health, and any subjects with abnormalities were excluded, such as schizoaffective disorders, dementia, neurodegenerative and neurological disorder, cardiovascular disease, cerebrovascular disease, infections, cancer, diabetes, hypertension, hyperlipidemia, and pregnant. Neither depressive patients nor healthy controls were experiencing drug or alcohol abuse/dependence, which was determined by the laboratory urine tests.

Informed consent was obtained before subjects were recruited. This protocol employed was approved by the Clinical Research Ethics Committee of the Affiliated Guangji Hospital of Soochow University, and all experiments were carried out in accordance with the approved guidelines and regulations.

### Clinical Variables

A detailed questionnaire including a complete medical history, physical examination, and medical and psychological conditions was obtained from all subjects. Additional information was collected from available medical records.

Cognitive functions were measured using the RBANS (Form A)^52^. The RBANS was composed of 12 subtests that were used to calculate 5 age-adjusted index scores and a total score. The test indices were immediate memory (composed of listing learning and story memory tasks), attention (composed of digit span and coding tasks), language (composed of picture naming and semantic fluency tasks), visuospatial/constructional (composed of figure copy and line orientation tasks), and delayed memory (composed of list recall, story recall, figure recall, and list recognition tasks). The RBANS was previously translated into Chinese, and its clinical validity and test-retest reliability were established in healthy controls and psychiatric disorder in our previous study^53^. To ensure consistency and reliability of rating, the two clinical psychologists simultaneously attended a training session for standardizing their use of the RBANS prior to the start of the study. Thereafter, they maintained an intraclass correlation coefficient of 0.93 on the RBANS at the repeated assessments.

### Measurement of Serum ApoB Levels

Serum samples from 70 depressive patients were collected between 7 and 9 AM following an overnight fast. HITACHI 7180 automatic biochemistry analyzer (Hitachi High-Technologies Corporation, Japan) was used for measuring serum ApoB levels by immunoturbidimetric method using a commercially available kit (Medical System Biotechnology, Ningbo, China). A full description of this method has been reported in a previous study^54^. Serum samples were assayed by a technician blind to the clinical situation. Each assay was run in duplicate. Inter- and intra-assay variation coefficients were 4% and 1.5%, respectively.

### Statistical Analysis

Group comparisons on the clinical and demographic data used Student’s *t*-tests or analysis of variance (ANOVA) for continuous variables and chi-square for categorical variables. The RBANS total and index scores of cognitive functions were compared between depressive patients and healthy controls using ANOVA. When the significant differences in cognitive functions were found between two groups using ANOVA, multivariate ANOVA (MANOVA) will further analyze these differences in cognitive functions, with gender, age, education, BMI, smoking, and suicide status as the covariates. Bonferroni corrections were applied to each test to adjust for multiple testing. Relationships between serum ApoB levels and the scores of cognitive deficits of depressive disorder were measured with Pearson’s product moment correction coefficients. Stepwise multivariate analysis using the scores of cognitive deficits of depressive disorder as dependent variables was used to investigate the impact of serum ApoB levels and other variables including gender, age, education, BMI, smoking and suicide status, age of illness onset, age of first hospitalization, duration of illness, number of hospitalization, the types of antidepressants, SDS and SAS standard score. SPSS version 17.0 was used to do all statistical analysis. Continuous data were presented as Mean ± SD and all p values were 2 tailed at the significance level of <0.05.

## Additional Information

**How to cite this article**: Hui, L. *et al*. Serum ApoB levels in depressive patients: associated with cognitive deficits. *Sci. Rep.* **7**, 39992; doi: 10.1038/srep39992 (2017).

**Publisher's note:** Springer Nature remains neutral with regard to jurisdictional claims in published maps and institutional affiliations.

## Figures and Tables

**Table 1:** Clinical and demographic characteristics in depressive patients and healthy controls.

Variables	Depressive Patients (n = 90)	Healthy Controls (n = 90)	F or χ^2^	*P*-value
Gender (male/female)	30/60	30/60	0.00	1.00
Age (years)	34.98 ± 10.78	34.98 ± 10.70	0.00	1.00
Education (years)	10.12 ± 3.34	9.74 ± 3.55	0.57	0.45
BMI (kg/m^2^)	21.62 ± 3.16	24.13 ± 3.70	23.72	**<0.001**
Smoking (smoker/nonsmoker)	8/82	26/64	11.75	**<0.001**
Suicide (attempter/no-attempter)	56/34	0/90	81.29	**<0.001**
Age of Illness Onset (years)	31.30 ± 10.23			
Age of First Hospitalization (years)	33.67 ± 10.54			
Duration of Illness (months)	50.70 ± 90.31			
Number of Hospitalizations	0.96 ± 0.92			
Types of Antidepressants				
SNRI	20 (22.22%)			
SSRI	53 (58.89%)			
Never Taking Antidepressants	17 (18.89%)			
SDS Standard Score	61.77 ± 12.73			
SAS Standard Score	52.0 ± 12.14			
Apolipoprotein B (g/L)	0.86 ± 0.21			

Mean ± SD (standard deviation); BMI = body mass index; SNRI = serotonergic and noradrenergic reuptake inhibitor; SSRI = selective serotonergic reuptake inhibitor; SDS: self-rating depression scale; SAS: self-rating anxiety scale.

**Table 2:** Comparisons of total and index scores of the RBANS between depressive patients and healthy controls.

RBANS Score	Depressive Patients (n = 90)	Healthy Controls (n = 90)	F	*P*-value^a^	*P*-value^b^ [Corrected]
Immediate Memory	75.92 ± 42.67	80.57 ± 17.21	3.81	0.053	0.318
Attention	92.82 ± 14.65	92.30 ± 18.44	0.04	0.837	1.000
Language	76.70 ± 15.25	96.36 ± 13.57	58.21	**<0.001**	**<0.001**
Visuospatial/Constructiona	85.29 ± 15.74	80.94 ± 14.37	0.84	0.361	1.000
Delayed Memory	77.43 ± 19.02	95.97 ± 54.73	7.72	**0.006**	**0.036**
Total Score	75.17 ± 15.15	84.73 ± 13.15	19.56	**<0.001**	**<0.001**

The nominally significant *P*-values (p < 0.05) are showed in bold.

^a^*P*-values were analyzed by controlling for gender, age, education, BMI, smoking and suicide status.

^b^*P*-values were further adjusted by Bonferroni correction.
